# Role of Ketogenic Diets in Neurodegenerative Diseases (Alzheimer’s Disease and Parkinson’s Disease)

**DOI:** 10.3390/nu11010169

**Published:** 2019-01-15

**Authors:** Dariusz Włodarek

**Affiliations:** Department of Dietetics, Faculty of Human Nutrition and Consumer Sciences, Warsaw University of Life Sciences (WULS-SGGW), 159c Nowoursynowska Str., 02-776 Warsaw, Poland; dariusz_wlodarek@sggw.pl; Tel.: +48-225937024; Fax: +48-22-5937028

**Keywords:** ketogenic diet, neurodegenerative disease, Alzheimer disease, Parkinson disease

## Abstract

The goal of this review was to assess the effectiveness of ketogenic diets on the therapy of neurodegenerative diseases. The ketogenic diet is a low-carbohydrate and fat-rich diet. Its implementation has a fasting-like effect, which brings the body into a state of ketosis. The ketogenic diet has, for almost 100 years, been used in the therapy of drug-resistant epilepsy, but current studies indicate possible neuroprotective effects. Thus far, only a few studies have evaluated the role of the ketogenic diet in the prevention of Parkinson’s disease (PD) and Alzheimer’s disease (AD). Single studies with human participants have demonstrated a reduction of disease symptoms after application. The application of the ketogenic diet to elderly people, however, raises certain concerns. Persons with neurodegenerative diseases are at risk of malnutrition, while food intake reduction is associated with disease symptoms. In turn, the ketogenic diet leads to a reduced appetite; it is not attractive from an organoleptic point of view, and may be accompanied by side effects of the gastrointestinal system. All this may lead to further lowering of consumed food portions by elderly persons with neurodegenerative diseases and, in consequence, to further reduction in the supply of nutrients provided by the diet. Neither data on the long-term application of the ketogenic diet in patients with neurodegenerative disease or data on its effects on disease symptoms are available. Further research is needed to evaluate the suitability of the ketogenic diet in the therapy of AD- or PD-affected persons.

## 1. Introduction

The first reports of studies indicating that the fasting or fasting-mimicking diet may have a beneficial effect on certain medical conditions were published at the beginning of the 20th century. The ketogenic diet (KD) was first used in order to reduce the incidence of epileptic seizures [1,2] and, over time, its application in other diseases was studied, including such medical conditions as amyotrophic lateral sclerosis (Charcot’s disease) [3,4], traumatic brain injury [5], cerebral ischemia [6], and neurodegenerative diseases (Parkinson’s disease and Alzheimer’s disease [7]. The use of KD enables fasting mimicking. This diet is characterized by a high fat content, few carbohydrates, and normal protein content. Whereas carbohydrates constitute approximately 55% of the energy value in the traditional diet, with approximately 30% of fat and 15% of protein, these proportions in classical KD are up to 8% for carbohydrates, 90% for fat, and approximately 7% for protein [8,9,10]. The most frequent KD form includes mainly long-chain fatty acids (which was proposed by Doctor Wider in 1921 to treat children with drug-resistant epilepsy. In Dr. Wilder’s diet, fats provided approximately 90% of energetic diet value, with very limited volumes of carbohydrates and protein) [10]. Although the diet proved to be an effective therapy for epilepsy in a considerable number of patients, adherence to the diet’s rules may pose a number of problems. To follow KD, it is necessary to introduce drastic changes in eating habits, which are unpalatable and difficult to maintain, especially from a long-term perspective. These problems may particularly be a concern for teenagers, adults, and families with many children, as well as individuals who are not fond of such a diet. Thus, a more attractive variety of this diet has been devised that allows users to achieve a similar metabolic effect. A diet based on medium-chain triglycerides (MCT) was applied as a form of therapy in the 1950s [11], which led to an increase in the concentration of ketone bodies in the blood, even if carbohydrates were present in the diet; thus, this version of KD enables the production of ketone bodies without drastic changes in diet composition [12]. However, its use is associated with the occurrence of dyspeptic conditions, limiting its more widespread application. In addition, it is not more effective than the traditional KD. Another version of KD was a combination of the traditional diet version and an MCT-based diet, but its effectiveness was similar to that of the traditional KD [11]. Another form of KD is a modified version based on the Atkins diet, which was proposed in 2003. In this version, carbohydrates are limited to up to 5% of energy in the diet, whereas neither the general energetic value of the diet nor its protein content undergoes any modification [10]. See Table 1 for the characteristics of various forms of KD. 

It must be noted that patient compliance with the KD (even modified) is poor, due to the restrictive nature of the diet and symptoms related to gastrointestinal adversity. Various ketone sources exist naturally in food; dairy products are a natural source of β-hydroxybutyrate [13]. Some studies suggest that an exogenous ketone supply increases the level of ketone bodies in serum [14,15].

Stubbes et al. [14] ordained 16 healthy non-obese volunteers following an overnight fast. Subjects consumed 395 mg/kg ketone ester on an empty stomach or immediately following a standard meal. The serum BHB (beta-hydroxybutyrate) level was tested one hour after administration of ketone ester. Serum BHB was lower in the group that consumed the standard meal versus the “empty stomach” group (2.1 mM ± 0.2 mM versus 3.1 mM ± 0.1 mM). These extreme doses convert to 31.6 grams of ketone ester for an 80 kg person, and were well-tolerated. In another study [15] using an oral dose of (R)-3-hydroxybutyl (R)-3-hydroxybutyrate, a monoester of the BHB molecule was quantified at 714 mg/kg body weight. This high dose was administered for five days, three times per day. The maximum plasma ketones were achieved within 2 h (3.30 mM BHB and 1.19 mM acetoacetate). These doses convert to 57.1 grams of ketone ester for an 80 kg (176 lb) person, and were well-tolerated also. 

Very promising data about the ketogenic diet comes from an observation by Murray et al. [16] of rats. They investigated the effects of the novel ketone ester diet on the physical and cognitive performance of animals. In the novel ketone ester diet, 27% of energy came from protein, 39% from carbohydrates, and 34% from total fat, but the diet was supplemented with (R)-3-hydroksybutyl (R)-3-hydroksybutytate as 30% of calories. This is very interesting, because this proportion of macronutrients is similar to fat-rich Western diets. They concluded that the novel ketone diet improved physical and cognitive functions in animals, and its energy-sparing proportions suggest that it may help to treat a range of human conditions with metabolic abnormalities.

## 2. Search Strategy and Selection Criteria

The literature’s analyses on the ketogenic diet in the context of neurodegenerative diseases was based on the principles of a structured literature review, a tool used in developing evidence-based knowledge in a rigorous and relevant way [17]. Publications included in PubMed, the Cochrane Library, and Google Scholar were analyzed, including original research articles, meta-analyses, and systematic reviews. The searched terms were: “ketogenic diet”, “neurodegenerative disease”, “Alzheimer’s disease”, and “Parkinson’s disease”, in combination with specific terms, such as “metabolism”, “cognition”, “beta-hydroxybutyrate”, ”acetoacetate”, “ketogenic formula”, and “medium chain triglyceride” related to ketogenic diet complications and side effects. The review concentrated primarily on publications from the last 10 years, but also included highly regarded older publications.

## 3. Metabolic Changes Associated with the Use of the Ketogenic Diet 

In the course of the KD, the body modifies its metabolism to its fasting-related form. In the initial stage of the KD, blood glucose concentration lowers, and the insulin-to-glucagon ratio is decreased. A higher glucagon concentration leads to mobilization of glucose acquisition from its resources in the liver (from glycogen). Following two or three days of fasting or after a drastic reduction of carbohydrate supplementation in the diet (below 20 g/day), glycogenesis is suppressed and glucose reserves begin to be insufficient for the normal course of fat oxidation through the supply of oxaloacetate in the Krebs cycle [8], as well as to meet the demands of the Central Nervous System (CNS). Since CNS uses glucose as a primary energy source, after 2–3 days of fasting it has to identify an alternative source of energy, which is provided by the ketone bodies acetoacetate, 3-hydroxybutyrate, and acetone. The process of their production is called ketogenesis, and occurs mainly in the mitochondrial matrix of hepatic cells [18].

Acetoacetate is the main ketone body produced in the liver, while 3-hydroburate is found mainly in blood. The production of acetoacetic acid is, in normal conditions, of small significance, while the acid is metabolized by a number of tissues, e.g., muscular or cardiac tissue. In the case of overproduction, its portion is metabolized to the other ketone bodies. The blood concentration of ketone bodies is, in physiological conditions, very low (<0.3 mmol/L vs. glucose concentration (approx. 4 mmol/L)). When ketone bodies achieve concentrations above 4 mmol/L, they become a source of energy for the CNS. In the case of either fasting or a diet with very low carbohydrate content, glycemia, although decreasing, remains at its physiological level. In these conditions, the glucose is formed from two sources: glucogenic amino acids and glycerol, released from triglycerides. In the first days of fasting or the KD, the main source of glucose is via neoglucogenesis from amino acids. In the days following, the importance of glycerol as a glucose source increases progressively, while the contribution of amino acids decreases. 

The concentration of ketone bodies in persons with an undisturbed glucose metabolism reaches a maximum value of 8 mmol/L, while blood pH remains unchanged (not lower) at pH = 7.4 [18,19]. Ketosis, which occurs in persons without disturbances in their carbohydrate metabolism, resulting from fasting or a very low-carbohydrate diet, is a completely physiological mechanism. It was first referred to by Hans Krebs as “physiological ketosis” to differentiate it from the pathological ketoacidosis seen in the diabetes type 1 [20]. In the course of “physiological ketosis”, insulin and glucose concentrations decrease, while glucagon concentration increases in order to maintain blood glucose concentration at a normal level. In persons suffering from diabetes mellitus, diabetic acidosis may be identified, which is a pathological condition with concentrations of ketone bodies exceeding 25 mmol/L, resulting from an insulin deficit with a simultaneously increased glucose concentration (>300 mg/dL) and decreased blood pH (pH < 7.3), which may lead to death [18].

## 4. Ketogenic Diet Neuroprotective Activity

In neurodegenerative diseases, it has so far not been possible to propose any causative therapy, since the atiology of the diseases is still not well-understood. The goal of neuroprotection is then either a slowing down or a complete stop of the processes, which leads to neuronal death in the CNS [9]. 

Increased production of ketone bodies by the liver and reduction in blood glucose concentration are key factors of significance for the therapeutic effects of KD. An increased concentration of ketone bodies is largely a consequence of fatty-acid oxidation. Oxidative stress and mitochondrial dysfunction are highlighted as central features of brain degenerative diseases. Oxidative stress, a condition that occurs due to an imbalance in oxidant and antioxidant status, has been known to play a vital role in the pathophysiology of neurodegenerative diseases, including AD and PD. Although reactive oxygen species (ROS) play a pivotal role in several cellular and signaling pathways at physiological concentrations (e.g., cell cycle regulation, phagocytosis, and enzyme activation), excessive generation of ROS leads to several harmful effects, including DNA, lipid, and protein damage. Mitochondrial dysfunction and enhanced apoptosis, accompanied by a poor antioxidant status, are the mechanisms of AD pathogenesis. The pathology of PD is characterized by the gradual and selective loss of dopaminergic neurons in the substantia nigra pars compacta. Imbalance in dopamine metabolism due to oxidative stress has been recognized as a contributor to this disease [21]. 

The neuroprotective properties of the KD may be related to biochemical changes which occur in the body as a result of glycolysis inhibition and the increased formation and concentration of ketone bodies. Ketone bodies reveal neuroprotective effects by increasing mitochondrial respiration via an increase in ATP (adenosine triphosphate) production. It should be mentioned that β-hydroxybutyrate may provide more energy for the brain per unit of oxygen than glucose. Ketone bodies also reduce the production of free radicals by improving the efficiency of the mitochondrial respiratory chain complex (increasing NADH (nicotinamide adenine dinucleotide) oxidation and inhibiting mitochondrial permeability transition) [22]. Antioxidant action of KD results also from the increase in glutathione and glutathione peroxidase activity. It was observed in research on rats that the increase in antioxidant activity in the hippocampus was accompanied by an increase of glutathione peroxidase. It is suggested that the higher activity of this enzyme, induced by the ketogenic diet in the hippocampus, might contribute to the protection of this structure from neurodegenerative changes [23]. Another discussed mechanism of KD action is the ability to abate apoptosis. In addition, they control the stabilization of nerve-cell synapse functions. Moreover, the selected polyunsaturated fatty acids (e.g., arachidonic acid, docosahexaenoic acid, and eicosapentaenoic acid) may promote excitability of neuron-cell membranes while reducing inflammatory conditions and suppressing the production of free radicals. [24]. 

Another biochemical feature of the KD is the decrease in glycolytic flux linked with the decrease of the energy value during the days following the diet. Reduction of glycolysis is an essential feature of calorie restriction which has been shown to prolong the lifespan of numerous species, including primates. Although the direct link between caloric restriction and KD mechanisms raises certain controversies, both treatments result in a reduction of the blood glucose concentration, likely involving reduced glycolytic flux [24].

Additionally, the restriction of calories may exert a neuroprotective effect by improving mitochondrial functions, leading to the reduction of reactive oxygen species (ROS) production and increased energy output, as well as by decreasing inflammatory and pro-apoptotic activities and increasing levels of neuroprotective factors, such as neurotrophins (brain-derived neurotrophic factor (BDNF), neurotrophin-3–NT-3, glial cell line-derived neuritrophic factor (GDNF)) and molecular chaperones (proteins that prevent aggregation of polypeptides into potentially toxic components). Caloric restriction also has anti-inflammatory effects; it can reduce levels of NFĸB, the central component of the inflammatory process, and block the synthesis of interleukins (IL1β, IL2, IL4, IL6) and tumor necrosis factors (TNFα) and suppresses the activity of cyclooxygenase-2 (COX-2) and inducible nitric oxide synthase (iNOS) [25].

## 5. Ketogenic Diet and Alzheimer’s Disease

Epidemiological studies demonstrate that a diet rich in saturated fatty acids increases the risk of AD [26]. What is more, a fat-rich diet, applied in transgenic mice, may accelerate cognitive impairment by promoting systemic inflammation, enhancing oxidative stress, and aggravating neuronal apoptosis [27,28]. At the same time, those studies did not assess the effects of the KD, a diet supplying big fat volumes.

Studies carried out on an animal model of Alzheimer’s disease indicate a possible beneficial effect of the KD for this medical condition. The KD was found to reduce the volumes of solved amyloid-beta in homogenates of murine brains [29]. It was also observed that a long-term administration of ketone body esters to mice improved their cognitive functions and reduced beta-amyloid and highly phosphorylated tau proteins in the brain [30]. The benefits of the KD was also proven with regard to the motor functions of the experimental animals, with no effect on either amyloid-beta or protein tau deposits [31,32]. Ma et al. [33] investigated the influence of the ketogenic diet on the neurovascular function and alteration of gut microbiomes in young healthy mice. They observed that this diet may enhance brain vascular function, increase beneficial gut microbiota, and improve metabolic profile (e.g., reduce blood glucose levels and body weight, and increase blood ketone levels). This intervention may also reduce the risk of AD. Studziński et al. [34], in their studies on senile dogs, demonstrated that a diet with MCTs improved mitochondrial function by control of the oxidation process. They also identified a trend in the observed decrease of amyloid beta concentrations in the brain. 

The studies also demonstrated a potential impact of KD on the course of Alzheimer’s disease in humans. Reger et al. [35] concluded that oral administration of MCT led to the elevation of plasma ketone body levels, and that it may improve cognitive functioning in older adults with memory disorders. In their study, 20 subjects with AD or mild cognitive impairment consumed a drink either containing emulsified MCTs or that which was a placebo. Significant increases in levels of the ketone body β-hydroxybutyrate were observed 90 min after treatment when cognitive tests were administered. The effects of MCTs administration were moderated by the apolipoprotein E (APOE) genotype. On cognitive testing, MCT treatment facilitated performance on the Alzheimer’s Disease Assessment Scale-Cognitive Subscale (ADAS-cog) for ε4− subjects, but not for ε4+ subjects. Higher ketone values were associated with greater improvement in paragraph recall with MCT treatment relative to the placebo across all subjects. 

Henderson et al. [12] similarly administered medium-chain triglycerides to subjects with mild and moderate AD. The study was randomized, placebo-controlled, and double-blinded. The included participants received one dose of the agent during the first 7 days of the study, followed by two doses (20 g of MCT in total) administered at breakfast from day 8 to day 90. The administration of that type of fatty acid led to increased serum concentrations of ketone bodies and improved cognitive functioning. It should, however, be noted that no such effect was observed in subjects with the APOEε4 genotype. 

Ota et al. [36] administrated medium-chain triglycerides to 20 Japanese patients with mild-to-moderate AD on separate days (50 g of a ketogenic formula containing 20 g of MCTs or an isocaloric placebo formula without MCTs). Patients took the ketogenic formula daily for up to 12 weeks and underwent neurocognitive tests 120 minutes after intake of the formula. The patients’ plasma levels of ketogenic bodies were increased 120 min after intake of the formula. After 8 weeks, the patients showed significant improvement in their immediate and delayed logical memory tests compared to their baseline score. At 12 weeks they showed significant improvement in the digit-symbol coding test and immediate logical memory tests compared to the baseline. 

In the Ketogenic Diet Retention and Feasibility Trial [37], 15 patients with AD maintained an MCT-supplemented KD (approximately 70% of energy as fat, including the MCT; 20% energy as protein; and less than 10% of energy as carbohydrates). MCT oil contained a combination of C8 and C10:0 fatty acids. In general, the MCT dosage provided approximately 10% of total energy from fat during the first week and increased by increments of 10% during each successive week until reaching 40%. They observed that in completely achieved ketosis, the mean of the Alzhiemer’s Disease Assessment Scale cognitive subscale score improved significantly during the diet and reverted to baseline after the washout. 

Mild cognitive impairment (MCI) is a dementia-preceding condition and the first manifestation of neurodegeneration in persons with Alzheimer’s disease. Krikorian et al. [38] randomly applied a high-carbohydrate (50% of energy from carbohydrates) or low-carbohydrate diet (5–10% of energy from carbohydrates, i.e., 20–50 g of carbohydrates daily) in 23 subjects. After 6 weeks of the intervention, the authors observed improved verbal memory performance for the subjects on the low-carbohydrate diet. The levels of ketone bodies were positively correlated with memory performance. It should, however, be emphasized that the low-carbohydrate diet had much lower energetic value than the high-carbohydrate diet (~1000 kcal vs. ~1600 kcal), and was associated with a considerable decrease of insulin level in the blood. The authors concluded that even short-term use of a low-carbohydrate diet could improve memory function in older adults with an increased risk for AD. Although the observed effect may be attributable in part to correction of hyperinsulinemia, other mechanisms associated with ketoses, such as reduced inflammation and enhanced energy metabolism, also may have contributed to the improvement in neurocognitive functioning. 

More evidence regarding the influence of the KD on AD will be available after publication of reports from ongoing registered randomized clinical trials sponsored by Johns Hopkins University (NCT02521818), Wake Forest University (NCT03130036, NCT03472664 and NCT02984540), Universite de Sherbrooke (NCT02709356), and the University of British Columbia (NCT02912936) [39]. In these studies, participating patients with subjective memory impairment, mild AD, and/or healthy controls were used to evaluate the impact of 6–18 weeks of the modified Ketogenic-Mediterranean diet to a low-fat diet; 12 weeks of the modified Atkins diet compared to the recommended diet for seniors to achieve a healthy eating index; 1 month treatment with the two different medium-chain triglycerides oil emulsions (40–60 oil or C8 oil), or 10 days, twice a day, and supplementation with a lactose-free skim milk drink containing either 10–50 g/day of MCT oil or 10–50 g/day of placebo (high-oleic sunflower oil).

Another possible influence of KD on the course of dementia and Alzheimer’s disease is also discussed. Studies indicate that an increased dietary supply of unsaturated fatty acids (especially polyunsaturated fatty acids and omega 3) may result in a lower risk of this condition [40,41,42]. It is then possible that the beneficial effect of KD on the amelioration of Alzheimer’s disease is associated with a higher supply of unsaturated fatty acids vs. a normal or high-carbohydrate diet [11].

## 6. The Ketogenic Diet and Parkinson’s Disease

In Parkinson’s disease (PD), dopaminergic neurons in the substantia nigra are affected by a degeneration process leading to motor and non-motor disturbances. Animal and in vitro studies have demonstrated a beneficial effect of ketone bodies on the course of PD. 1-methyl-4-phenyl-1,2,3,6-tetrahydropyridine (MPTP) produced the death of dopaminergic substantia nigral cells, both in vitro and in vivo, producing a syndrome indistinguishable from Parkinson’s disease. It was shown that beta-hydroxybutyrate acts in vitro as a neuroprotective agent against the toxicity of MPTP on dopaminergic neurons [7]. In addition, Tieu et al. [43] concluded that in mice administered with the MPTP neurotoxin, which induces a defect of the mitochondrial complex I, an administration of beta-hydroxylactate reduced the neurotoxicity of the compound via effects exerted on complex II and by improvement of cellular respiration and ATP production. Improved motor skills were also seen in the experimental mice, together with an increased dopamine volume in the mesencephalon. Shaafi et al. [44] observed in a rat model of Parkinson’s diseases a beneficial influence of the KD on the motor function of the experimental animals. Moreover, a combination of the diet with pramipexole increased the efficacy of the medicinal product. Joniec-Maciejak et al. [45] examined the possible protective effects of octanoic acid in the mouse model of PD induced by MPTP. They observed that the administration of octanoic acid led to inhibition of the neurodegenerative processes seen after MPTP administration. The results suggested that a probable mechanism of the neuroprotective action of octanoic acid is related to an increase in metabolic activity in striatal mitochondria. 

In vitro studies also revealed a potentially advantageous influence of ketone bodies on the functionality of synapses in an induced dysfunction of the respiratory mitochondrial chain, caused by an administration of rotenone, being an inhibitor of complex I and of the 3-nitropropionic acid, which is an inhibitor of complex II. The protective effects of ketone bodies could result from an antioxidative activity, improved ATP synthesis, and from the effect on the ATP-sensitive potassium channel (KATP) [46,47]. In other studies, the beneficial effects of ketone bodies were described, resulting from a mitigated inflammatory condition induced by MPTP administration [48] and from reduced apoptosis of dopaminergic cells exposed to substances causing their death [49]. In a study on a rat model of Parkinson’s disease, Cheng et al. [50] concluded that KD, via glutathione activity, exerted a neuroprotective effect against the toxic effect of 6-hydroxydopamine.

In a clinical study, Vanitallie et al. [51] observed 5 patients with PD who had agreed to adhere to KD rules in their home environments. The observation continued for 28 days. The researchers observed some improvement in the scores of the Unified Parkinson’s Disease Rating Scale (UPDRS), while not excluding the possibility of placebo effects. It is worth emphasizing that in the KD applied in the study, the contribution of particular components to the energetic value of the diet was 90% for fat, 2% for carbohydrates, and 8% for protein. In a habitual diet, the proportion of energy from proteins is higher, while a low-protein diet improves the bioavailability of levodopa. Four of the studied patients received levodopa. Therefore, when evaluating the effects of KD on improved UPDRS scores in their respective cases, one should also take into account the possible effect of protein supply. Very interesting data came from the research of Phillips et al. on this topic [52]. They assessed the effect of a low-fat versus ketogenic diet in 47 patients with Parkinson’s disease (38 individuals completed the study). The low–fat diet provided 1750 kcal per day, composed of 42 g of fat (approximately 22% of energy), 75 g of proteins (17% of energy), 246g of carbohydrates (56% of energy), and 33 g of fiber (5%). The ketogenic diet provides 1750 kcal per day, composed of 152 g of fat (approximately 78% of energy), 75 g of protein (17% of energy), 16g of carbohydrates (3–5% of energy), and 11 g of fiber (1–5% energy). For those with higher energy needs, a calorie-booster was prepared with an adequate diet proportion of macronutrients. The diets were followed for eight weeks. Both diet groups showed significantly improved motor and nonmotor symptoms; however, the ketogenic group showed greater improvements in nonmotor symptoms.

## 7. Adverse Effects of the Ketogenic Diet

A major problem in long-term use of the KD is the adherence of patients to dietary recommendations. High volumes of fat-rich components in the diet (cheeses, eggs, butter, oils, meat, etc.) may lead to poor tolerance. Side effects may thus include nausea, vomiting, constipation, and lower appetite. In addition, dehydration, hepatitis, pancreatitis, hypoglycemia, hypertriglyceridemia, hyperuricemia, hypertransaminemia, hypercholestorolemia, hypomagnesemia, and hyponatremia are among the adverse effects of the KD. Late effects may include reduced mineral bone density, nephrolithiasis, cardiomyopathy, deficits of vitamins and mineral components, impaired hepatic functions, neuropathy of the optic nerve, anemia, and constipation or enhanced atherosclerosis [9]. 

KD, as a fat-rich and low-carbohydrate diet, is associated with a certain restriction of the energetic value of dietary portions, and leads to metabolic effects which eventually reduce body weight [53]. Although the anorectic activity of KD is commonly reported, it still remains unexplained as to which mechanisms mediate the process. The anorectic effects of KD may, among others, result from an increased concentration of long-chain fatty acids, which reduces the expression of neuropeptide Y (NPY) in the hypothalamus, while NPY is an appetite-stimulating factor. Ketosis leads to postprandial secretion of cholecystokinin-pancreozymin, a hormone responsible for the motor function of the gastric system (it inhibits the motor function of the stomach) and downregulating consumed food volumes, food portion sizes, and meal duration times. Moreover, the concentration of circulating ghrelin decreases, where ghrelin is a food intake-controlling hormone. It is worth pointing out that ketosis may also exert orexigenic effects by increasing the circulating levels of adiponectin, increasing brain gamma-aminobutyric acid (GABA) and AMP-activated protein kinase (AMPK) phosphorylation, and decreasing brain ROS production. However, the net balance between contrasting stimuli cause a loss of appetite and, in consequence, reduced food intake [54]. 

Persons suffering from neurodegenerative diseases are at high risk for malnutrition. This results from disease symptoms which may bring about lower food intake, among other reasons. In Parkinson’s disease, the reception (feeling) of sensory sensations is compromised, and there are difficulties regarding the preparation and consumption of meals associated with motor disorders. There are also problems with chewing and swallowing, a rapid feeling of satiety, and others. In Alzheimer’s disease, patients may demonstrate disturbances in the senses of taste and smell, neurological disorders in the character of apraxia, behavioral disturbances during eating, dysphagia, and many other symptoms which impede food consumption. Therefore, the application of DK in persons with neurodegenerative diseases may raise certain doubts, as their nutritional condition is an important factor influencing the life expectancy of the patients. The few studies so far which have been undertaken with the participation of KD-using patients were relatively short in their duration. Therefore, findings on the effects of long-term KD application on the nutritional condition of patients and on the course of their disease are yet to be discovered.

In order to maintain proper blood glucose concentration levels in the course of KD application, the process of gluconeogenesis is enhanced, among others, by an increased secretion of glucocorticoids, glucagon, and the growth hormone. All this results in the increased catabolism of proteins and the use of glucogenic amino acids as glucose precursors. In consequence, there is an increased secretion of metabolism nitrogen products, so diuresis in enhanced to maintain a constant dilution of these metabolites in urine [55], while in persons with normal kidney function, it need not exert any negative effects on the body [55]. However, it may be a problem for persons with renal impairment to excrete larger volumes of nitrogen metabolism waste products. 

Sarcopenia is a problem which affects elderly persons, and its incidence increases with age [56]. It also concerns persons with neurodegenerative diseases. A proper supply of protein is a necessary condition to maintain muscle mass. According to current recommendations, elderly people should consume 1.0–1.2 g of protein/kg per day, or even more if they are physically active [57]. KD, especially when the diet energy value is decreasing, may lead to a general supply of protein that is too low, although its contribution to the diet energy value may be normal or even higher than recommended. Such a situation may lead to catabolism of structural proteins (especially in muscles). Long-term use of KD may also bring about catabolism and reduced synthesis of functional proteins (e.g., enzymes, membrane proteins) [53]. Unfortunately, it may be rather difficult to achieve an appropriate supply of energy and protein in the diet of persons on the KD, taking into account its appetite-reducing effects and lower organoleptic attractiveness. Energetic deficits and insufficient protein volumes in a diet may have serious consequences for health if dietary restrictions, resulting from KD rules, are applied for a longer period of time.

## 8. Conclusions

The available results of research projects dealing with the use of the KD and ketone bodies in neurodegenerative diseases are fairly promising. At the same time, the majority of these studies were employed in vitro or by using animal models. The number of studies with human participation is rather small, and those that exist feature relatively short therapy duration periods. It is rather difficult to say how significant this therapeutic approach may be in the future, especially because the use of the diet alone is difficult and concerns elderly people with possible concomitant diseases which may impose certain constraints on the possibility of KD application. Further studies are necessary, especially for research of long-term KD effects on the symptoms and course of neurodegenerative diseases, as well as on the general well-being of affected patients.

## Figures and Tables

**Table 1:** Characteristics of ketogenic diets [10].

Macronutrients	Classical Ketogenic Diet	MCT-Based Diet	Modified Atkins Diet
	Energy (%)		
Fat	90	70	70
Protein	7	10	25
Carbohydrates	8	20	5
